# Effects of qigong exercise on cardiovascular risk factors in patients with metabolic syndrome: A systematic review and meta-analysis

**DOI:** 10.3389/fphys.2023.1092480

**Published:** 2023-02-24

**Authors:** Shuoxiu Tao, Zaimin Li

**Affiliations:** ^1^ Institute of Physical Education, Chengdu University, Chengdu, China; ^2^ Institute of Physical Education, Guizhou University of Finance and Economics, Guiyang, China

**Keywords:** qigong exercise, metabolic syndrome, systematic review, meta-analysis, random control trails

## Abstract

**Background:** As a traditional Chinese exercise system, Qigong includes many types of exercises, including Baduanjin, Wuqinxi, Yijinjing, and Liuzijue. However, reviews highlighting the effects of a specific type of Qigong exercise in patients with metabolic syndrome or risk factors for metabolic syndrome are limited, and no articles have systematically evaluated the effects of Qigong exercise on cardiovascular risk factors in patients with metabolic syndrome. Therefore, this systematic review aimed to evaluate the effects of Qigong exercise on cardiovascular risk factors in patients with metabolic syndrome.

**Objective:** Relevant randomized controlled trials were identified to conduct a meta-analysis of the effects of Qigong exercise on patients with metabolic syndrome, and to further explore the overall impact, heterogeneity, and publication bias related to the effects of Qigong exercise on metabolic syndrome.

**Methods:** We searched for RCTs of Qigong exercise in patients with metabolic syndrome from the following databases: Pubmed, Web of Science, The Cochrane Library, Scopus, Embase, Physiotherapy Evidenced Database (PEDro), Google Scholar, Chinese Biomedical Literature Database (CBM), Chinese National Knowledge Infrastructure Database (CNKI), Chinese Science, Wanfang Data, and the VIP database. The search duration was set from the establishment of the database to 16 April 2022. We used the “Bias Risk Assessment” tool recommended by Cochrane Manual 5.0 to assess the methodological quality of the included literature and the R (version 3.6.2) package gemtc to analyze the data.

**Results:** A total of seven RCTs with 486 participants were included in the meta-analysis. The results showed that Qigong exercise had significant effects on waist circumference (standardized mean difference [SMD] = −0.67; 95% CI, −1.16 to −0.17), systolic blood pressure (standardized mean difference = −0.53; 95% CI, −0.78 to −0.28) and triglyceride level (SMD = −0.60; 95% CI, −0.79 to −0.41). Subgroup analyses showed that 6-month Qigong exercise significantly improved diastolic blood pressure (SMD = −1.06; 95% CI, −1.57 to −0.56), high-density lipoprotein cholesterol level (SMD = 1.45; 95% CI, 1.06–1.85), total cholesterol level (SMD = −0.65; 95% CI, −1.04 to −0.27), and body mass index (SMD = −0.97; 95% CI, −1.23 to −0.72). For fasting blood glucose (SMD = −1.12; 95% CI, −1.58 to −0.67), the effect of a 3-month intervention seemed more effective than 6 months of Qigong exercise, but the evidence was insufficient. In addition, Qigong exercise had minimal effects on low-density lipoprotein cholesterol levels (SMD = −1.22; 95% CI, −1.95 to −0.50).

**Conclusion:** Qigong may be an alternative exercise mode to improve cardiovascular risk factors in patients with metabolic syndrome. However, the findings are limited by the number and quality of the included studies, and require validation through more high-quality studies.

## 1 Introduction

Metabolic syndrome (MetS) is a disease mainly caused by a sedentary lifestyle, high levels of subjective stress, and an inadequate diet, which mainly causes central obesity, elevated blood pressure, elevated blood glucose levels, and/or dyslipidemia (Huang et al., 2018). It is an important risk factor for cardiovascular and cerebrovascular diseases, diabetes, chronic kidney disease and other diseases (Song, 2005) and a major health problem worldwide. According to the study by Huang et al., the prevalence of MetS in China increased from 8.8% in 1991% to 29.3% in 2015 (Huang et al., 2018). Park and Kim reported that approximately 25% of Korean adults have MetS (Park and Kim, 2015). In the US, approximately 35% of adults meet the diagnostic criteria of MetS, and the prevalence rate is close to 50% in people over 60 years of age (Ford et al., 2002). Cardiovascular risk factors include overweight/obesity, hypertension, hyperglycemia and dyslipidemia, which can cause cardiovascular disease. Cardiovascular disease (CVD) is a broad term covering disorders of the heart and blood vessels and includes hypertension, coronary heart disease, cerebrovascular disease, peripheral vascular disease, heart failure, rheumatic heart disease, congenital heart disease, and cardiomyopathies (World Health Organization, 2017). MetS has been confirmed to increase the risk of CVD (Ford, 2005; Gami et al., 2007). Conversely, CVD is the main cause of death in most patients with MetS. A systematic evaluation of the global disease burden in relation to mortality and causes of death from 1990 to 2013 in the journal Lancet showed that MetS was characterized by obesity, hypertension, hyperglycemia and dyslipidemia, which also constituted the risk factors of MetS, and could easily progress to CVD.

Considering the CVD susceptibility of MetS patients, and the existence of multiple risk factors for CVD in patients with MetS, studies have increasingly focused on the effect of exercise training on cardiovascular risk factors in patients with MetS. Farinha JB pointed out that physical exercise may have a significant effect on weight loss and decreased glucose, cholesterol, and triglyceride levels and reduced levels of oxidative stress and proinflammatory markers in patients with MetS (Farinha et al., 2015). Hahn et al. (2009) found that 2 h/week of aerobic exercise can effectively delay the development of the disease in high-risk elderly patients with MetS. Miyatake et al. (2007) pointed out that moderate-intensity physical activity can reduce the incidence of MetS by about 2/3rd. Armin et al. reported that under conditions of diet control, an exercise intervention performed three times a week and involving 30 min of walking and 15 min of resistance exercise could significantly improve the risk components of MetS in middle-aged men (Heufelder et al., 2009). At the same time, Ostman conducted a systematic review of the research on the use of sports training to improve the symptoms of MetS patients, and found that exercise training improves body composition, cardiovascular, and, metabolic outcomes in people with MetS (Ostman et al., 2017). As a traditional sport in China, Qigong exercise focuses on mind, body, psychology and behavior, including breathing and physical exercise. Qigong exercise includes many types of exercise types such as “Baduanjin,” “Yijinjing,” “Liuzijue,” and “Wuqinxi,” which are characterized by gentle and slow movements (low-to-medium intensity aerobic exercise) and the coordination of body movements and breathing (Li et al., 2001). In comparison with other forms of sports, Qigong exercise is easy to learn and practice and does not have high equipment and space requirements (Li et al., 2019). This form of exercise has recently received attention in the scientific literature. Previous systematic evaluations reported that Qigong exercise was beneficial in improving health and treating chronic disease (Gouw et al., 2019; Lee et al., 2022), e.g., by improving motor function, depression, and quality of life in patients with Parkinson disease (Song et al., 2017), reducing stress and anxiety (Wang et al., 2014), ameliorating depression and other psychological diseases (Zou et al., 2018), and also reducing blood pressure levels (Lee et al., 2007).

A large amount of studies have also shown that Qigong exercise can influence MetS (Liu, 2012; Shi et al., 2013; Zou et al., 2013; Sun, 2015; Thongthawee et al., 2017; Ke et al., 2020; Jin et al., 2021). However, a unified understanding of the influence of Qigong exercise on cardiovascular risk factors in patients with MetS is currently unavailable, so the potential beneficial effects of Qigong exercise in improving cardiovascular risk factors in patients with MetS remain unclear. Therefore, we conducted a systematic review on this topic to determine insights that could facilitate decision-making and for clinical guidance for Qigong exercise prescriptions for patients with MetS.

## 2 Methods

This systematic review was planned and conducted in accordance with the Preferred Reporting Items for Systematic Reviews and Meta-analyses (PRISMA) guidelines (Picot et al., 2012) and the Cochrane Handbook of Systematic Reviews (Higgins et al., 2019).

### 2.1 Search strategies

The following electronic databases were searched by one reviewer (TSX) from their inception through 16 April 2022: Pubmed, Web of Science, The Cochrane Library, Scopus, Embase, Physiotherapy Evidenced Database (PEDro), Google Scholar, Chinese Biomedical Literature Database (CBM), Chinese National Knowledge Infrastructure Database (CNKI), Chinese Science, Wanfang Data, and the VIP database. The database searches were performed using a combination of key words and subject headings, namely, “Qigong, Baduanjin, Yijinjing, Wuqinxi, Liuzijue, mind-body exercise” and “metabolic syndrome” and “cardiovascular disease,” and adapted for each database as necessary. Taking the Web of Science as an example, the detailed research strategy was “Qigong” OR “Baduanjin” OR “Wuqinxi” OR “Liuzijue” OR “Yijinjing” OR “mind body exercise” AND “metabolic syndrome” AND “cardiovascular disease”. And it was presented in Table 1.

**TABLE 1:** Web of Science search strategy.

Order	Search terms
#1	Qigong
#2	Baduanjin
#3	Wuqinxi
#4	Liuzijue
#5	Yijinjing
#6	Mind body exercise
#7	#1 OR #2 OR #3 OR #4 OR #5 OR #6
#8	metabolic syndrome
#9	cardiovascular disease
#10	#7 AND #8 AND #9

### 2.2 Eligibility criteria

The inclusion criteria for this systematic review were based on the PICO framework (Higgins et al., 2019) and were as follows: 1) participants: studies on adult patients (more than 40–70 years of age) diagnosed with MetS; 2) intervention events: Qigong exercise was conducted in the intervention group; 3) control group: the control group consisted of individuals who did not perform exercise or did not perform Qigong exercise; 4) outcome measurements: the study outcome measurers mainly included body mass index (BMI), waist circumference (WC), systolic blood pressure (SBP), diastolic blood pressure (DBP), and fasting blood glucose (FBG), triglyceride (TG), high-density lipoprotein cholesterol (HDL-C), low-density lipoprotein cholesterol (LDL-C), and total cholesterol levels (TC); 5) design: only randomized controlled trials (RCTs) were eligible.

### 2.3 Exclusion criteria

The following studies were excluded: 1) studies in which the intervention method is not clear; 2) articles that have been repeatedly published; 3) reviews, observational studies, abstract articles, and non-RCT studies were unqualified.

### 2.4 Data extraction and quality assessment

Titles and abstracts were screened and selected independently by two reviewers (TSX and LZM), and potentially eligible articles were read in full by the two reviewers. Disagreements were settled by a third reviewer. The reviewers collected the following information: 1) literature information, including the authors’ names, year of publication, and country; 2) sample size and adherence; 3) study settings; 4) intervention programs; 5) duration, time, and frequency of the intervention; 6) outcome measurements, including those identified as CVD risk factors and potential CVD risk factors: BMI, WC, SBP, DBP, FBG, TG, HDL-C, LDL-C, TC; 7) mean age (years) in the intervention and control groups; and 8) adverse effects.

The methodological quality assessment of the included studies was performed using the “Bias Risk Assessment” tool recommended by Cochrane Manual 5.0 to evaluate the quality of the included studies in terms of the random allocation method, allocation concealment, blinding method, the integrity of the result data, and the selective reporting of bias of the study results. For each study, these six items were evaluated as “yes” (low bias), “no” (high bias) or “unclear” (lack of relevant information or uncertainty of bias). The quality of the methodology included in the study was independently assessed by two expert group members, and any differences were resolved by a third reviewer.

### 2.5 Statistical analysis

The R 3.6.2 software gemtc package was used for data processing. For binary variables, OR was used as the efficacy analysis statistic, and for numerical variables, the standardized mean difference (SMD) was used as the efficacy analysis statistic. Each effect was expressed in terms of the 95% confidence interval (95% CI). Because the number of included articles was only seven, to ensure the validity of the statistical evaluations, the random-effects model was used for meta-analysis of all indicators. The heterogeneity of the included studies was evaluated using *p*-values (threshold point, 0.1) and I^2^ statistics (I^2^ ≤ 50%, low heterogeneity; I^2^ > 50%, high heterogeneity) analysis (Hanley et al., 2003). When the heterogeneity was greater than 50%, subgroup analysis was performed to determine the source of heterogeneity. Notably, the intervention time and frequency showed no significant differences, so subgroup analysis was performed only for the possible heterogeneity factor of intervention duration. In addition, in our systematic review, sensitivity analysis was conducted to assess the robustness of the findings of the meta-analysis.

## 3 Results

### 3.1 Study selection

**FIGURE 1:**
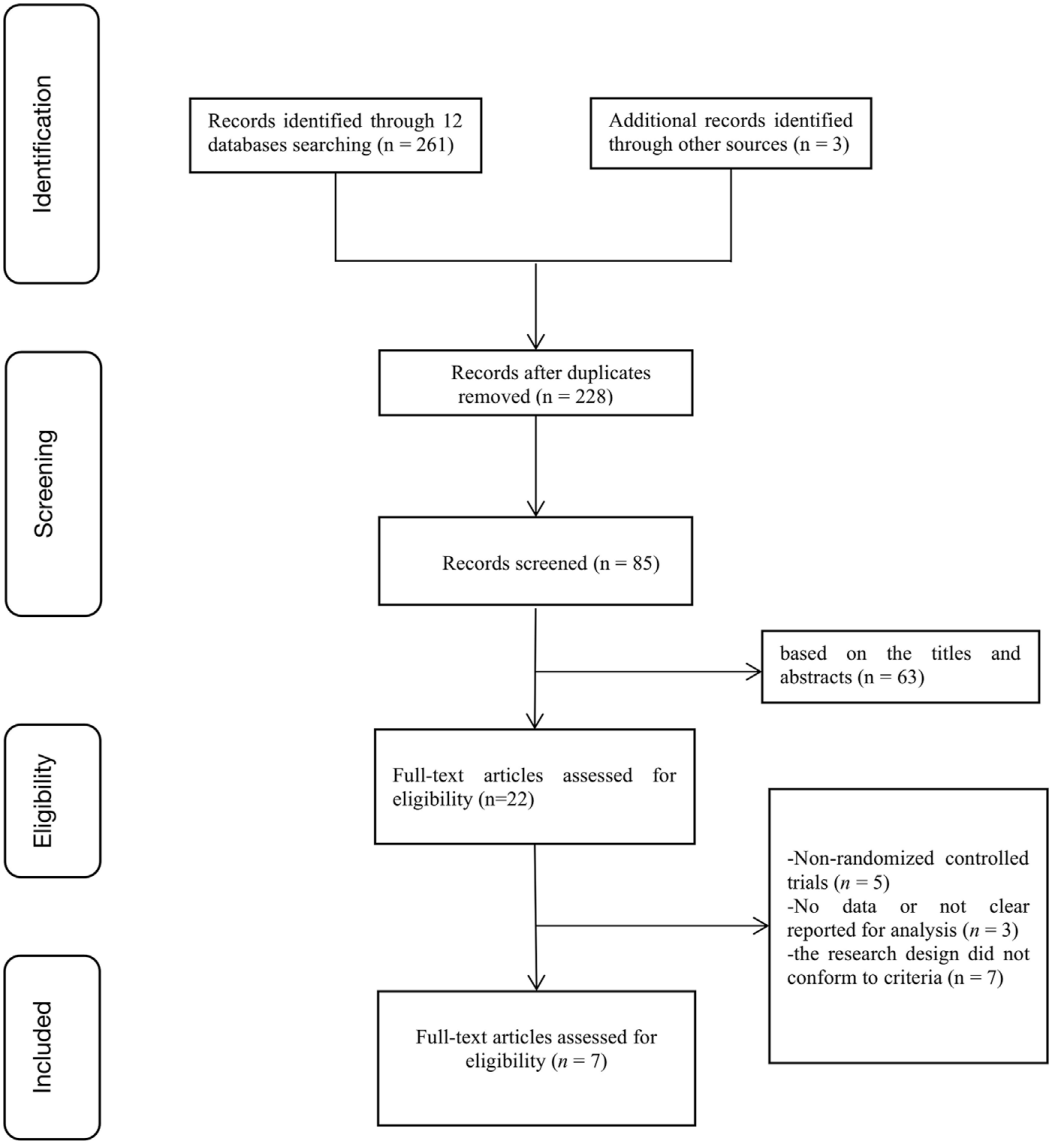
Flowchart outlining the study selection.

A total of 264 articles were identified from the databases. After discarding duplicate studies and reading the titles and abstracts of the remaining studies, 22 articles were selected. After reading the full text of these 22 articles, we removed non-RCTs (n = 5), studies with no or unclear outcome measures (n = 3), and studies using research designs that did not conform to the inclusion criteria (n = 7). The remaining seven eligible studies were included in our meta-analysis (Figure 1).

### 3.2 Characteristics of the included studies

The seven RCTs included a total of 486 participants (251 in the experimental group and 235 in the control group). Six studies were conducted in China, while the seventh was conducted in Thailand. Three studies involved analyses of community-dwelling individuals, two assessed inpatients or outpatients, while the other two did not mention the study setting. All articles mentioned that the baseline values for the test and control groups were similar and comparable. Randomization was mentioned in all studies. The retention rate of all studies was 100%. All experimental groups performed isolated exercise interventions. None of the selected studies reported adverse effects (Table 2).

**TABLE 2:** Baseline characteristics of the included studies.

Study ID	Country	Setting	Sample size	Completion n/total n	Adherence (%)	Groups	Intervention time and frequency	Intervention duration	Outcome measurements	Mean age (years) Intervention (SD); Control (SD)	Adverse effects reported
Shi-2013 Shi et al., (2013)	China	community-dwelling	T = 20 C = 20	40/40	100	Qigong Control	60-min sessions 7 times/week	12-week	BMI, FBG, TG, HDL-C, LDL-C, TC	56.7 ± 6.79, 56.87 ± 5.98	NR
Ke-2020, Ke et al., (2020)	China	Inpatients or outpatients	T = 21 C = 20	41/41	100	Qigong Control	30-min sessions 7 times/week	12-week	WC, SBP, DBP, FBG, TG, HDL-C, LDL-C, TC	42.21 ± 5.31, 42.18 ± 5.21	NR
Jin-2021, Jin et al., (2021)	China	Inpatients or outpatients	T = 45 C = 45	90/90	100	Qigong Control	60-min sessions 7 times/week	3-month	SBP, DBP, FBG, TG, HDL-C, LDL-C, TC	69.7 ± 11.2, 61.3 ± 10.6	NR
Liu-2012 Liu, (2012)	China	community-dwelling	T = 22 C = 18	40/40	100	Qigong Control	60-min sessions 6 times/week	6-month	BMI, WC, SBP, DBP, FBG, TG, HDL-C, LDL-C, TC	68.17 ± 3.50, 67.9 ± 5.00	NR
Sun-2015 Sun, (2015)	China	NR	T = 15 C = 15	30/30	100	Qigong Control	60-min sessions 5 times/week	6-month	BMI, SBP, DBP, FBG, TG, HDL-C, LDL-C, TC	40–50 (only report range)	NR
Zou-2013 Zou et al., (2013)	China	community-dwelling	T = 100 C = 100	200/200	100	Qigong Control	60-min sessions 7 times/week	6-month	BMI, FBG, TG, HDL-C, LDL-C, TC	57.42 ± 6.67, 57.52 ± 6.20	NR
Thongthawee -2016, Thongthawee et al., (2017)	Thailand	NR	T = 28 C = 27	55/55	100	Qigong Control	60-min sessions 4 times/week	12-week	BMI, WC, SBP, DBP	51.00 ± 2.56, 52.56 ± 2.56	NR

Note: T = trait group, C = control group, NR = not reported, N = number, WC: waist circumference, BMI = body mass index; SBP = systolic blood pressure; DBP = diastolic blood pressure; FBG = fast blood glucose; TG = triglyceride; HDL-C = high-density lipoprotein cholesterol; LDL-C = low-density lipoprotein cholesterol; TC = total cholester.

### 3.3 Methodological quality assessment

**FIGURE 2:**
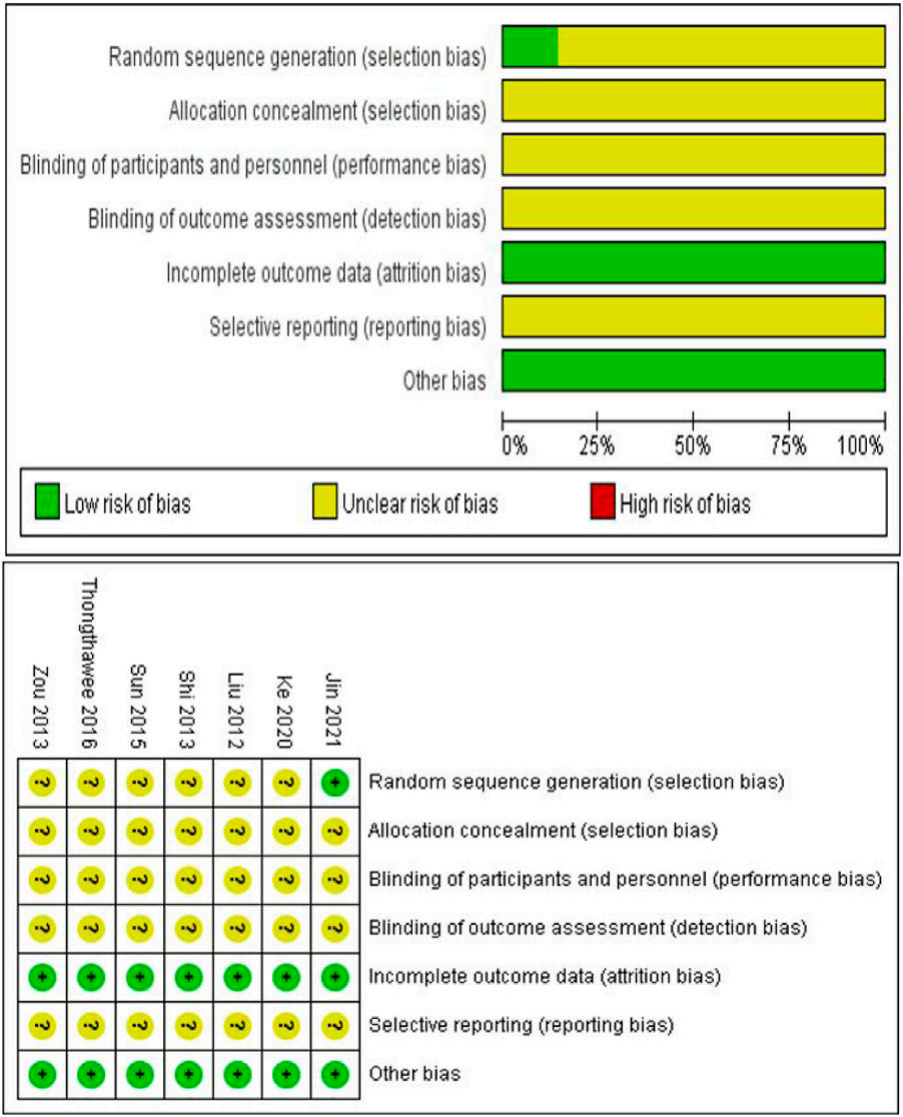
Assessment of the risk of bias based on the Cochrane Manual.

The quality of the included studies was scored according to the Cochrane risk of bias assessment tool using six evaluation criteria: Random sequence generation, allocation concealment, blinding method (blinding of participants and personnel, blinding of outcome assessment), incomplete outcome data, selective reporting, and other bias. The score for each criterion was categorized as low, high, and unknown risk, which were expressed in green, red, and yellow, respectively, and recorded as 1, 0, and 0.5 points, respectively. The total score was six points. Scores greater than 4 points indicated a low risk of research bias, while scores ≤4 indicated a high risk of research bias. All articles reported complete outcome data. All articles had other sources of bias, and only one article described the method of generating a random allocation sequence in detail. None of the articles clearly described allocation concealment, the specific methods for implementing blinding, and the approaches to avoid selective research reporting. The quality of the included articles in this study was mainly between 4 and 4.5 points, indicating a low risk of research bias (Figure 2).

### 3.4 Meta-analysis of outcome indicators

#### 3.4.1 Meta-analysis of the overall effect

**FIGURE 3:**
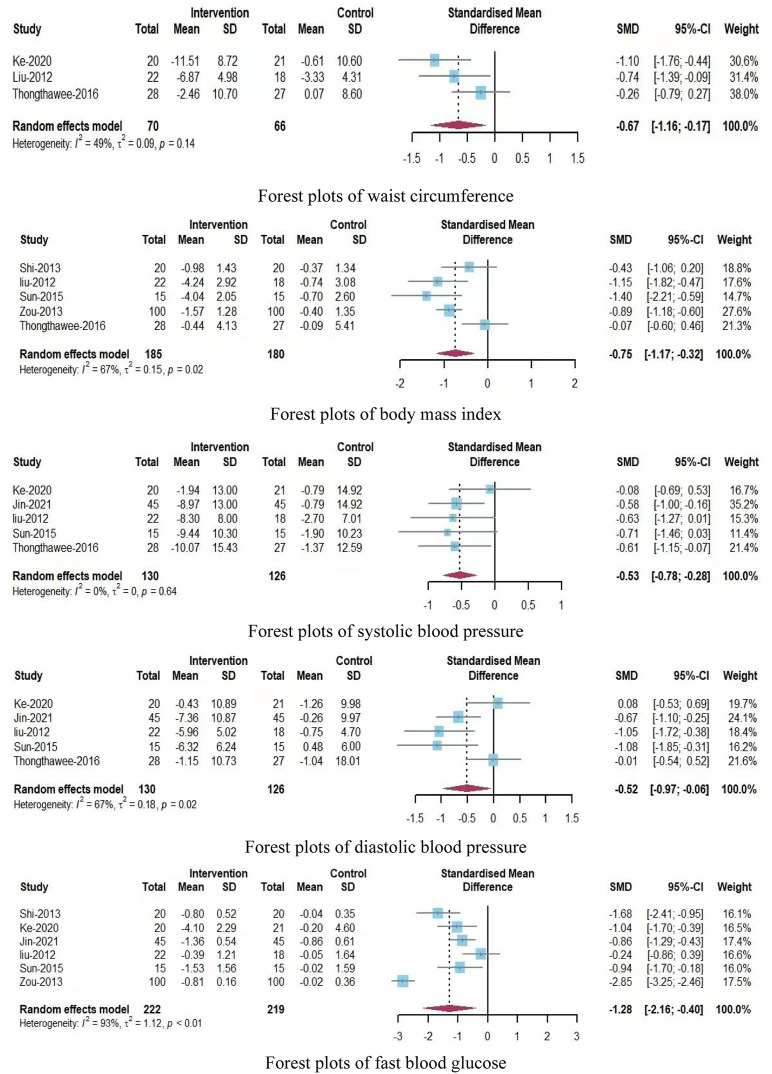
Forest plots of WC, BMI, SBP, DBP, and FBG level.

**FIGURE 4:**
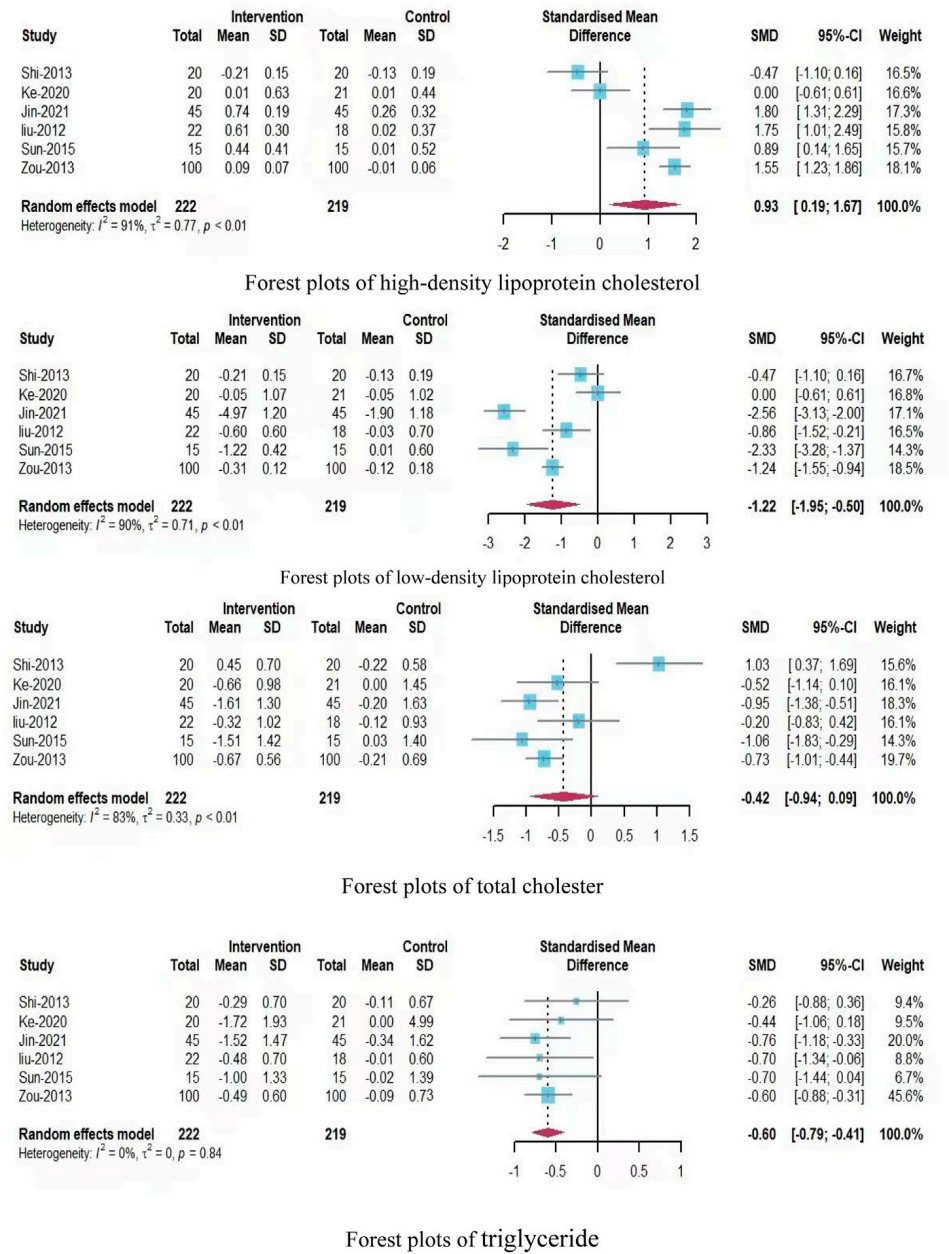
Forest plots of HDL-C, LDL-C, TC, and TG levels.

A total of nine outcome indicators were analyzed in this systematic review, and the forest plots showed the following findings (Figure 3; Figure 4):

(1) Assessments of waist circumference included three studies with 136 participants (70 participants in the experimental group and 66 participants in the control group). In comparison with the control group, the Qigong exercise group showed moderate heterogeneity (SMD = −0.67; 95% CI, −1.16 to −0.17; P = 0.14, I^2^ = 49%), indicating that Qigong exercise had a significant effect on WC and that there was no need to further assess the source of heterogeneity.
(2) Assessments of body mass index included five studies with 365 participants, (185 participants in the experimental group and 180 participants in the control group). In comparison with the control group, the Qigong exercise group showed high heterogeneity (SMD = −0.75; 95% CI, −1.17 to −0.32; P = 0.02; I^2^ = 67%), suggesting that Qigong exercise had a slightly significant effect on BMI and that the sources of heterogeneity required further assessment in a subgroup analysis.
(3) Assessments of systolic blood pressure included five studies with 256 participants (130 in the experimental group and 126 in the control group). In comparison with the control group, the experimental group showed low heterogeneity (SMD = −0.53; 95% CI, −0.78 to −0.28; P = 0.64; I^2^ = 0%), indicating that Qigong exercise had a moderate impact on SBP, and that there was no need to further discuss the source of heterogeneity.
(4) Assessments of diastolic blood pressure included five studies with 256 participants (130 in the experimental group and 126 in the control group). In comparison with the control group, the experimental group showed high heterogeneity (SMD = −0.52; 95% CI, −0.97 to −0.06; P = 0.02; I^2^ = 67%), indicating that Qigong exercise had little significant effect on DBP and that the source of heterogeneity required further evaluation in a subgroup analysis.
(5) Assessments of the fast blood glucose level were reported in six studies with 441 participants (222 in the experimental group and 219 in the control group). In comparison with the control group, the Qigong exercise group showed high heterogeneity (SMD = −1.28; 95% CI, −2.16 to −0.40; P < 0.01; I^2^ = 93%), indicating that Qigong exercise had a slightly significant effect on the FBG level and that the source of heterogeneity required further evaluation in a subgroup analysis.
(6) Assessments of the high-density lipoprotein cholesterol level were reported in six studies with 441 participants (222 in the experimental group and 219 in the control group). In comparison with the control group, the Qigong exercise group showed high heterogeneity (SMD = 0.93; 95% CI, 0.19–1.67; P < 0.01, I^2^ = 91%), indicating that Qigong exercise had a slightly significant effect on the HDL-C level and that the source of heterogeneity required further evaluation in a subgroup analysis.
(7) Assessments of the low-density lipoprotein cholesterol level were also reported in six studies with 441 participants (222 in the experimental group and 219 in the control group). In comparison with the control group, the Qigong exercise group showed high heterogeneity (SMD = −1.22; 95% CI, −1.95 to −0.50; P < 0.01; I^2^ = 90%), indicating that Qigong exercise had a slightly significant effect on the LDL-C level and that the source of heterogeneity required further evaluation in a subgroup analysis.
(8) Assessments of the total cholester level were also been reported in six studies with 441 participants (222 in the experimental group and 219 in the control group). In comparison with the control group, the Qigong exercise group showed high heterogeneity (SMD = −0.42; 95% CI, −0.94 to 0.09; P < 0.01; I^2^ = 83%), indicating that Qigong exercise had a slightly significant effect on the TC level and that the source of heterogeneity required further evaluation in a subgroup analysis.
(9) Assessments of the triglyceride level were performed in six studies with 441 participants, (222 in the experimental group and 219 in the control group). In comparison with the control group, the Qigong exercise group showed low heterogeneity (SMD = −0.60; 95% CI, −0.79 to −0.41; P = 0.84; I^2^ = 0%), indicating that Qigong exercise had a significant effect on the TG level and that there was no need to further discuss the source of heterogeneity.

#### 3.4.2 Sensitivity analysis

The sensitivity analysis was performed by excluding individual studies one-by-one to calculate the combined SMD value and 95% CI. The results showed no significant changes between the combined results of the remaining studies and the meta-analysis results, indicating that the original analysis results were not affected by specific studies and that the changes were indeed significant and thereby confirming that the meta-analysis results were relatively stable. Because of the large amount of data from all parameters, we only took SBP as an example in our research (Table 3).

**TABLE 3:** Sensitivity analysis of systolic blood pressure.

Outcome measurement	Study	SMD	95%CI
systolic blood pressure	Omitting Ke-2020	−0.62	−0.89; −0.34
	Omitting Jin-2021	−0.50	−0.81; −0.19
	Omitting Liu-2012	−0.51	−0.78; −0.23
	Omitting Sun-2015	−0.50	−0.77; −0.24
	Omitting Thongthawee-2016	−0.50	−0.79; −0.22
	Total	−0.53	−0.78; −0.22

#### 3.4.3 Subgroup analysis

Subgroup analysis was performed for outcome measurements showing high heterogeneity (I^2^ > 50) to further identify the source of heterogeneity. The indicators in subgroup analyses mainly include experimental country, participant age, intervention method, intervention setting, intervention time, intervention frequency, and intervention duration. In this review, the experimental countries (six studies in China, one in Thailand), participant age (average participant age range, 40–70 years; five articles assessed middle-aged participants, two studied elderly participants), intervention method (all articles evaluated Qigong exercise), intervention setting (two articles assessed inpatients or outpatients, three assessed community-dwelling individuals, and the remaining two did not report the study setting), intervention time (one article described 30-min sessions, and seven articles described 60-min sessions), intervention frequency (four articles described interventions performed seven times a week; while one article each described interventions performed four, five, or six times a week), and intervention duration (four articles described interventions performed for 3 months, while three described interventions performed for 6 months). Therefore, to ensure the accuracy and reliability of the findings, subgroup analysis was only conducted using the intervention duration. The subgroup analysis results (Table 4) were as follows: 1) For BMI, both 3-month (I^2^ = 0%) and 6-month (I^2^ = 0%) Qigong exercise were effective. 2) For the FBG level, based on the heterogeneity in the findings, 3-month Qigong exercise (I^2^ = 44%, *p* = 0.17) seemed more effective than 6-month Qigong exercise (I^2^ = 96%, *p* < 0.01). 3) For DBP, 6-month Qigong exercise (I^2^ = 0%, *p* = 0.95) was more effective than 3-month Qigong exercise (I^2^ = 64, *p* = 0.06). 4) For the HDL-C level, 6-month Qigong exercise (I^2^ = 33%, *p* = 0.23) was more effective than 3-month Qigong exercise (I^2^ = 95%, *p* < 0.01). 5) For the LDL-C level, both 3- and 6-month Qigong exercise did not show significant heterogeneity, but on the basis of the *p*-values, 6-month exercise (*p* = 0.04) was more effective than 3-month exercise (*p* < 0.01). 6) For the TC level, 6-month Qigong exercise (I^2^ = 39%, *p* = 0.19) was more effective than 3-month Qigong exercise (I^2^ = 92%, *p* < 0.01).

**TABLE 4:** Sub-group analysis of overall effect.

Sub-group	Duration (month)	SMD	95%CI	P	I^2^ (%)
body mass index	3	−0.22	−0.63;−0.18	0.39	0
	6	−0.97	−1.23;−0.72	0.43	0
fast blood glucose	3	−1.12	−1.58;−0.67	0.17	44
	6	−1.36	−3.12; 0.41	<0.01	96
diastolic blood pressure	3	−0.23	−0.74; 0.27	0.06	64
	6	−1.06	−1.57;−0.56	0.95	0
high-density lipoprotein cholesterol	3	0.45	−0.98; 1.89	<0.01	95
	6	1.45	1.06; 1.85	0.23	33
low-density lipoprotein cholesterol	3	−1.02	−2.61; 0.58	<0.01	95
	6	−1.37	−1.99; −0.75	0.04	68
total cholester	3	−0.16	−1.30; 0.97	<0.01	92
	6	−0.65	−1.04;−0.27	0.19	39

In general, for BMI and the HDL-C and TC levels, the longer the intervention duration, the better the effect. However, for the FBG level, the 3-month intervention seemed more effective. In addition, for the LDL-C level, Qigong exercise had no obvious improvement effect.

## 4 Discussion

The purpose of this systematic review was to analyze the influence of Qigong exercise on cardiovascular risk factors in patients with MetS. This systematic review of seven RCTs of Qigong exercise in patients with MetS provided evidence of the benefits of this exercise for improving the WC, SBP, and TG level. The subgroup analysis showed that a longer intervention (≥6 months) was more effective for the DBP and HDL-C and TC levels, but for the FBG level, the 3-month intervention had a better effect. For BMI, both 3- and 6-month interventions were effective, although the effect of 6-month exercise was better than that of 3-month exercise. In addition, this study also showed that Qigong exercise had little effect on the LDL-C level.

To our knowledge, no systematic evaluation and/or meta-analysis of the effects of Qigong exercise interventions on cardiovascular risk factors in patients with MetS has been conducted to date. However, some previous articles had similar themes. Zou et al. reviewed the effect of Wuqinxi on patients with cardiovascular disease risk factors of MetS, and found that exercise had a positive influence on SBP, DBP, and TC, TG, LDL-C, and HDL levels. In addition, their regression results showed that long-term Wuqinxi interventions significantly improved DBP and TC, TG, and LDL-L levels (Zou et al., 2019). Although the LDL-C results in our study were different from those in Zou’s study, the results for other indicators were the same or similar, the similarity was that for DBP, LDL-C, and TC, and the results of our study were obtained after a subgroup analysis to evaluate heterogeneity, further highlighting the reliability of our findings. Nevertheless, the reasons for the differences between our study and Zou’s study require explanation. In terms of participant inclusion, Zou’s study included “patients at risk of MetS” and “patients with MetS,” while our study specifically included “patients with MetS,” which directly led to the differences in the results. In addition, we also identified some studies on Taijiquan and yoga, which are similar to Qigong, and are also called mind–body exercise forms because of their similarities in movement characteristics (Wu et al., 2019), and their findings may further explain our results. Janita Pak Chun Chau et al. reviewed the research on the effects of Taijiquan on patients with MetS or adults at the risk of MetS. Of the 20 studies included in their article, one involved adults with MetS, and the other 19 involved adults with at least one MetS risk factor. Their systematic review found that Taijiquan could reduce WC (Chau et al., 2021), which was consistent with the results of our study. Another meta-analysis of the effects of yoga on patients with MetS showed that this exercise form had significant effects on WC and SBP, but had no significant effect on DBP and the TG, FBG, and HDL-C levels (Cramer et al., 2016), which was largely consistent with the results of our study and could also validate the reliability of the results of this study in some aspects.

This meta-analysis showed that Qigong exercise had significant effects on WC, SBP, and TG level, and the subgroup analysis confirmed the effects of Qigong exercise on BMI, DBP, and FBG, HDL-C, and TC levels. 1) The effects of Qigong exercise on WC and BMI may be attributable to the ability of Qigong exercise to dredge the meridians, promote blood circulation, regulate internal organs, and enhance health according to traditional Chinese medicine theory (Kong et al., 2022). The slow movements in Qigong exercise necessitate energy consumption during practice and thereby promote fat decomposition, which facilitates reductions in the WC and improvements in the BMI. 2) With regard to the effects of Qigong exercise on blood pressure indicators, blood pressure fluctuations are known to occur as a result of a complex interactions among the external environment and behavioral stimuli, internal cardiovascular regulation mechanisms, humoral influences, and rheological factors. Increasing evidence shows that high variability of blood pressure is a major vascular risk factor and a determinant of target organ damage (Divani et al., 2019; Tully et al., 2020). Qigong exercise can reduce the hyperactivity of the sympathetic nervous system and correct imbalances in the nervous system, which play important roles in blood pressure variability. In the subgroup analysis, the DBP-improving effect of 6-month Qigong exercise was better than that of 3-month Qigong exercise, which may be related to the short duration of exercise and lifestyle interventions and the lack of an understanding of the mechanisms underlying the effects of exercise. 3) The forest plot analysis did not show a significant effect of Qigong exercise on the FBG level, but the subgroup analysis indicated that 3 months of Qigong exercise had a greater effect on the FBG level than 6 months of Qigong exercise, contradicting the findings of previous studies in which prolonged Qigong exercise was shown to improve blood glucose indicators. This finding may be attributable to differences in the inclusion criteria among studies. Some studies only included adults living in communities, while our study included adults living in communities as well as hospitals. The training effects in different environments (Zhou et al., 2019), such as in a hospital environment, as well as the health status, health needs, and responses of individuals practicing Qigong exercise in these environments were clearly different from those in the community, which may have been responsible for the differences in results. Moreover, in the FBG subgroup analysis, the characteristics of the 3-month interventions were quite variable, with session durations of 30 and 60 min and exercise frequencies of 4 workouts a week and 7 workouts a week. In contrast, all interventions in the 6-month group were conducted in 60-min sessions with exercise frequencies of 5, 6, or 7 workouts a week. Proper exercise intensity may yield better health benefits from Qigong exercise. However, in the included studies of the 3-month subgroup, there were obvious differences in the time and duration of Baduanjin movement. This may be the reason why the exercise effect at 3 months was better than that at 6 months. Thus, more studies should be conducted to determine the effectiveness of Qigong exercise in controlling the FBG level. 4) In terms of the TG, HDL-C, LDL-C, and TC levels, the meta-analysis showed that Qigong exercise was more effective for the TG level, which may be because Qigong exercise may mobilize the body’s fatty acids to decompose TG, improve the activity of lipase, and reduce the serum lipid levels (Ke et al., 2020). In the subgroup analysis, for HDL-C and TC levels, 6-month Qigong exercise was more effective. However, Qigong exercise had no obvious effect on the LDL-C level.

The limitations of the study can be summarized as follows: 1) The quality scores of the included studies were 4–4.5 points, indicating a lack of high-quality literature. 2) Only seven RCTs were included in this meta-analysis, of which all except one study had less than 100 participants; thus, this meta-analysis may not have sufficiently addressed the risk of bias. Since only seven RCTs reported the effects of Qigong exercise in MetS patients, we cannot obtain accurate results for Qigong exercise in these patients. All articles mentioned randomization, but they did not elaborate on the specific method of randomization. Only one article mentioned allocation concealment without providing a detailed explanation, and the lack of information on allocation concealment may lead to systematic deviations of the treatment effect. 3) In the subgroup analysis, due to the lack of literature, only the intervention duration was analyzed, making it impossible to determine the influence of age, country, setting, intervention means, intervention time, and frequency on patients with MetS. 4) This review only included RCTs, highlighting the need to include diversified studies for overall evaluation. 5) All of the included studies were from Asia, including one study conducted in Thailand and six studies conducted in China. The lack of research in non-Asian populations reduced the representativeness of the findings. Moreover, the studies were published in English and Chinese, and the included research is incomplete.

## 5 Conclusion

This systematic review showed that Qigong exercise has positive effects on cardiovascular risk factors in MetS patients, especially on the WC, SBP, and TG level. However, this review showed no benefit of Qigong exercise on the LDL-C level in MetS patients. Moreover, considering the lack of relevant evidence, the effectiveness of Qigong exercise on FBG requires further discussion. Thus, the influence of Qigong exercise on the LDL-C and FBG levels in MetS patients needs to be investigated in more studies. As a traditional Chinese exercise form, Qigong exercise should be considered as an alternative exercise method for MetS. Future studies should aim to ensure allocation concealment and appropriate blinding to improve the experimental design. We should encourage the impact of long-term Qigong exercise on MetS patients, which provides a reference for improving various indicators of MetS patients’ Cardiovascular risk factors through Qigong exercise in the future.

## Data Availability

The original contributions presented in the study are included in the article/supplementary material, further inquiries can be directed to the corresponding author.
